# Piezo1 acts upstream of TRPV4 to induce pathological changes in endothelial cells due to shear stress

**DOI:** 10.1074/jbc.RA120.015059

**Published:** 2020-12-14

**Authors:** Sandip M. Swain, Rodger A. Liddle

**Affiliations:** 1 Department of Medicine, Duke University, Durham, North Carolina, USA; 2 Department of Veterans Affairs Health Care System, Durham, North Carolina, USA

**Keywords:** Piezo1, TRPV4, endothelial cell, shear stress, calcium elevation, adherens junctions, phospholipase A2, actin remodeling, 5′,6′-EET, 5′,6′-epoxyeicosatrienoic acid, AJs, adherens junctions, GSK101, GSK1016790A, HC067, HC067047, HUVEC, human umbilical vein endothelial cell, PLA2, phospholipase A2, PVP, pulmonary venous pressure, TRPV4, transient receptor potential vanilloid subfamily 4

## Abstract

The ion channels Piezo1 and TRPV4 have both, independently, been implicated in high venous pressure– and fluid shear stress–induced vascular hyperpermeability in endothelial cells. However, the mechanism by which Piezo1 and TRPV4 channels execute the same function is poorly understood. Here we demonstrate that Piezo1 regulates TRPV4 channel activation in endothelial cells and that Piezo1-mediated TRPV4 channel opening is a function of the strength and duration of fluid shear stress. We first confirmed that either fluid shear stress or the Piezo1 agonist, Yoda1, led to an elevation in intracellular calcium ([Ca^2+^]_i_) and that application of the Piezo1 antagonist, GsMTx4, completely blocked this change. We discovered that high and prolonged shear stress caused sustained [Ca^2+^]_i_ elevation that was blocked by inhibition of TRPV4 channel opening. Moreover, Piezo1 stimulated TRPV4 opening through activation of phospholipase A2. TRPV4-dependent sustained [Ca^2+^]_i_ elevation was responsible for fluid shear stress–mediated and Piezo1-mediated disruption of adherens junctions and actin remodeling. Blockade of TRPV4 channels with the selective TRPV4 blocker, HC067047, prevented the loss of endothelial cell integrity and actin disruption induced by Yoda1 or shear stress and prevented Piezo1-induced monocyte adhesion to endothelial cell monolayers. These findings demonstrate that Piezo1 activation by fluid shear stress initiates a calcium signal that causes TRPV4 opening, which in turn is responsible for the sustained phase calcium elevation that triggers pathological events in endothelial cells. Thus, deleterious effects of shear stress are initiated by Piezo1 but require TRPV4.

Endothelial cells are under constant mechanical force due to blood pressure and flow in both the arterial and venous systems. Blood flowing across endothelial cells within blood vessels generates shear stress. Under normal physiological conditions, a dynamic balance between mechanical shear stress and biological responses maintains endothelial integrity (1, 2, 3, 4, 5). Shear stress induces the release of endothelial vasodilatory factors such as nitric oxide, prostacyclin, and cytochrome *P-450* metabolites of arachidonic acid that are required for surrounding smooth muscle relaxation (6, 7, 8). Endothelial Ca^2+^–mediated mechanosensing is required for normal flow–mediated dilation (4, 5). Perturbation of endothelial shear stress that occurs with hypertension or excessive flow leads to vascular remodeling through the disruption of cytoskeletal proteins and vascular dysfunction involving the loss of endothelial integrity, increased endothelial cell stiffness, altered vasorelaxation properties, and leukocyte adhesion (2, 3, 9, 10, 11, 12, 13).

Clinically, high venous pressure is a major cause of pulmonary edema and mortality in patients with congestive heart failure (10, 11). Elevated vascular pressure can lead to endothelial barrier disruption and hyperpermeability due to loss of adherens junctions (AJs) between endothelial cells (3, 11). It has recently been demonstrated that the mechanosensitive ion channel Piezo1 mediates pressure-induced disruption of AJs and endothelial barrier breakdown in pulmonary vessels (11, 14). Piezo1 is activated by cell membrane tension caused by high pressure, shear stress, and membrane stretching which allows the influx of cations, mainly Ca^2+^, and triggers downstream calcium signaling (15, 16, 17, 18). These processes are important for maturation of the vasculature as deletion of Piezo1 impaired vascular development in mice and also blocked sprouting angiogenesis in response to shear stress (16). However, endothelial Piezo1 mediates pathological responses to pressue and is involved in atherosclerosis progression and inflammatory signaling (19).

Like Piezo1, the endothelial cell–expressed, calcium-permeable transient receptor potential vanilloid subfamily 4 (TRPV4) channel is expressed in various tissues and cells that are also pressure-sensitive (*e.g.*, vascular endothelium, urinary bladder, and airway epithelium) (9, 20, 21, 22). TRPV4 has been linked to several physiological functions including epithelial ciliary activity, regulation of blood flow, and shear-induced vasodilation and angiogenesis and pathological processes that involve endothelial dysfunction and actin disruption (4, 23, 24, 25). Blockade of TRPV4 channels protects against pulmonary edema and hyperpermeability induced by high pressure (10). Experimentally it has been shown that TRPV4 channels are activated by high shear stress, membrane stretching, and hypotonic cell swelling although they do not have true mechanoreceptor properties (4, 7, 26, 27, 28, 29, 30, 31). Therefore, how TRPV4 in endothelial cells senses physical force is not fully understood.

It was demonstrated previously that blood flow–mediated shear stress activates phospholipase A2 (PLA2), generating 5′,6′-epoxyeicosatrienoic acid (5′,6′-EET) from arachidonic acid (6, 28). Importantly, 5′,6′-EET has the ability to activate TRPV4. However, how shear stress activates PLA2 in endothelial cells is unknown.

We recently observed that stimulation of Piezo1 in pancreatic acinar cells is responsible for pressure-induced pancreatitis (32) and is linked to TRPV4 (33). Therefore, we postulated that Piezo1 signaling coupled to TRPV4 activation may also account for the effects of shear stress on endothelial cells. Here we demonstrate that high shear stress activates Piezo1 and causes an initial increase in [Ca^2+^]_i_ that triggers activation of TRPV4 in human umbilical vein endothelial cells (HUVECs) and HEK293T cells expressing both Piezo1 and TRPV4. Activation of TRPV4 causes a sustained [Ca^2+^]_i_ elevation leading to loss of endothelial cell contacts, actin disruption, and endothelial cell monocyte adhesion.

## Results

### Fluid shear stress–induced [Ca^2+^]_i_ overload is force and time dependent

**Figure 1:**
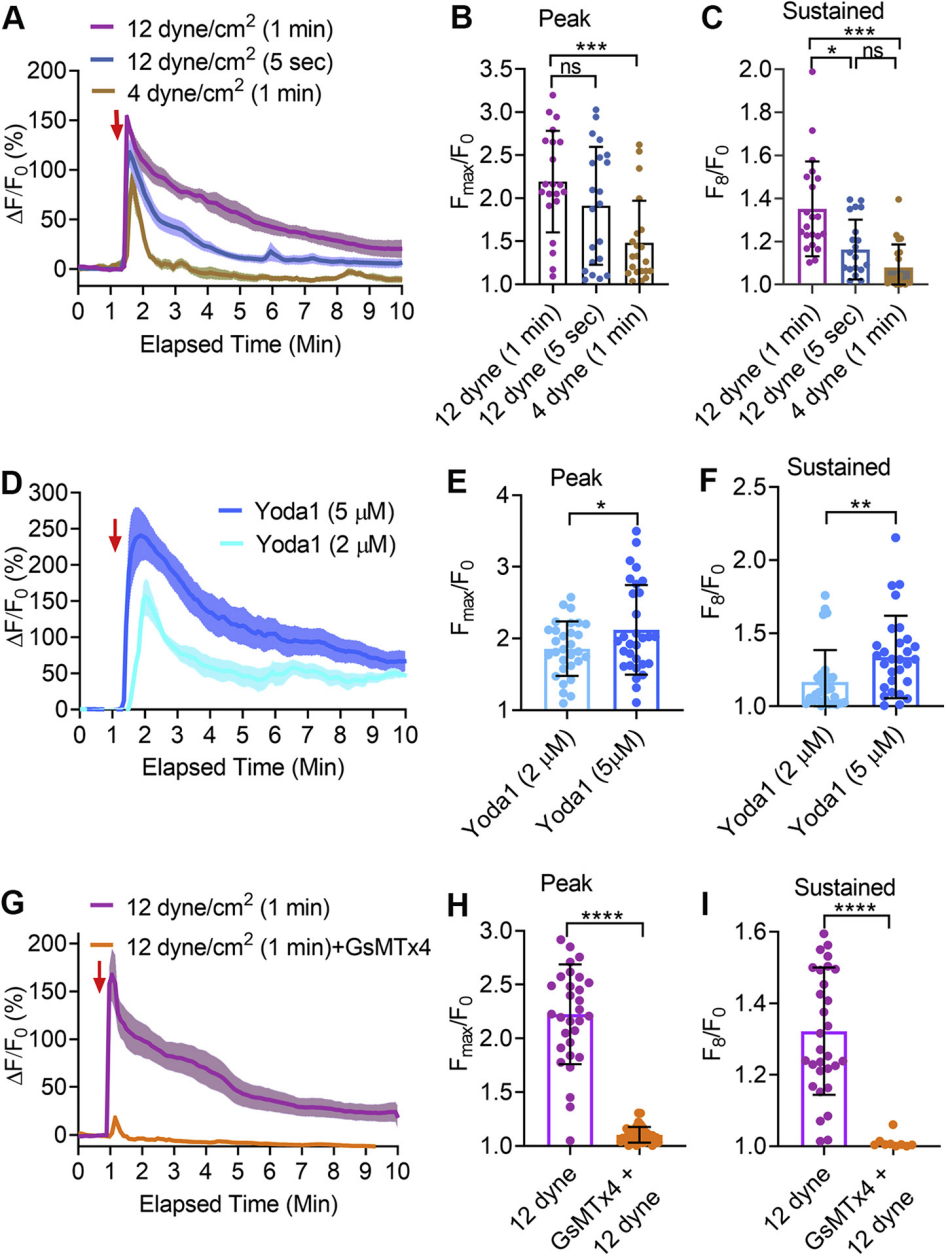
**Fluid shear stress–induced and Yoda1-induced [Ca**^**2+**^**]**_**i**_**elevation in HUVECs.***A*, the relative fluorescence intensities (ΔF/F_0_) of calcium 6-QF–loaded HUVECs are shown in response to applied shear stress at 12 dyne/cm^2^ or 4 dyne/cm^2^ for 1 min or 5 s. *B*, the graphs show the average maximum peak [Ca^2+^]_i_ intensity (F_max_/F_0_) and (*C*) the average peak intensity calculated at 8 min after initiation of force from 21 cells. *D*–*F*, Yoda1-induced [Ca^2+^]_i_ elevation in HUVECs. *D*, the relative fluorescence intensity (ΔF/F_0_) of calcium dye over time with Yoda1 (2 μM) or Yoda1 (5 μM). *E*, the graphs show the average maximum peak [Ca^2+^]_i_ intensity and (*F*) the average [Ca^2+^]_i_ intensity peak calculated at 8 min after the initiation of force from 30 cells. *G*–*I*, the effects of GsMTx4 on fluid shear stress–induced [Ca^2+^]_i_ elevation. GsMTx4 was applied 2 min before shear stress. *G*, the relative fluorescence intensity (ΔF/F_0_) of calcium dye over time with shear stress (12 dyne/cm^2^ for 1 min) with or without GsMTx4 (10 μM). *H*, the average maximum peak [Ca^2+^]_i_ intensity and (*I*) the average [Ca^2+^]_i_ intensity peak calculated at 8 min after initiation of force from 31 cells. Statistical analyses were performed using two-tailed Student’s *t* test, ∗*p* ≤ 0.05; ∗∗*p* ≤ 0.01; ∗∗∗*p* ≤ 0.001; ∗∗∗∗*p* ≤ 0.0001. Data are shown as mean ± SD. HUVEC, human umbilical vein endothelial cell.

Shear stress is a physiological activator of mechanical ion channels in endothelial cells. In endothelial cells, shear stress regulates levels of intracellular calcium ([Ca^2+^]_i_) and downstream calcium signaling (24, 34, 35, 36). We evaluated the magnitude and duration of shear stress forces that affected [Ca^2+^]_i_ in HUVECs by applying shear stresses at physiological level (≤5 dyne/cm^2^) and pathological levels (37, 38). Shear stresses of 4 and 12 dyne/cm^2^ applied for 1 min increased peak [Ca^2+^]_i_ (Fig. 1, *A*–*B*). Applying a force of 12 dyne/cm^2^ for 1 min caused a sustained elevation in [Ca^2+^]_i_ (intensity calculated at 8 min after initiation of force), but the same force applied for a shorter time (12 dyne/cm^2^ for 5 s) or lower shear stress (4 dyne/cm^2^) for 1 min elicited only a transient [Ca^2+^]_i_ rise and did not produce prolonged elevation in [Ca^2+^]_i_ (Fig. 1, *A*–*C*).

Endothelial cells express the mechanically sensitive, calcium-permeable ion channel Piezo1 (14, 17). As an initial step in evaluating its role, we utilized the Piezo1 agonist, Yoda1. Yoda1 (2 μM and 5 μM), in a dose-dependent manner, increased peak [Ca^2+^]_i_ and caused sustained [Ca^2+^]_i_ elevations (fluorescence intensity calculated at 8 min after Yoda1 application) (Fig. 1, *D*–*F*). Thus, the level of [Ca^2+^]_i_ produced by Piezo1 activation in HUVECs was dependent upon the Yoda1 concentration (Fig. 1, *D*–*F*). To determine if Piezo1 was responsible for shear stress–induced calcium influx, we used the mechanoreceptor blocker, GsMTx4 (39). GsMTx4 (10 μM) completely inhibited the shear stress (12 dyne/cm^2^)–induced elevation in [Ca^2+^]_i_ (Fig. 1, *G*–*I*). We observed that a higher dose of Yoda1 (10 μM) or GsMTx4 (10 μM) applied for up to 11 min did not affect the cell viability as all cells treated with Yoda1 or GsMTx4 responded to the calcium ionophore, ionomycin (1 μM) (Fig. S1, *A*–*D*).

### TRPV4 is responsible for the Piezo1-induced sustained [Ca^2+^]_i_ elevation in HUVECs

**Figure 2:**
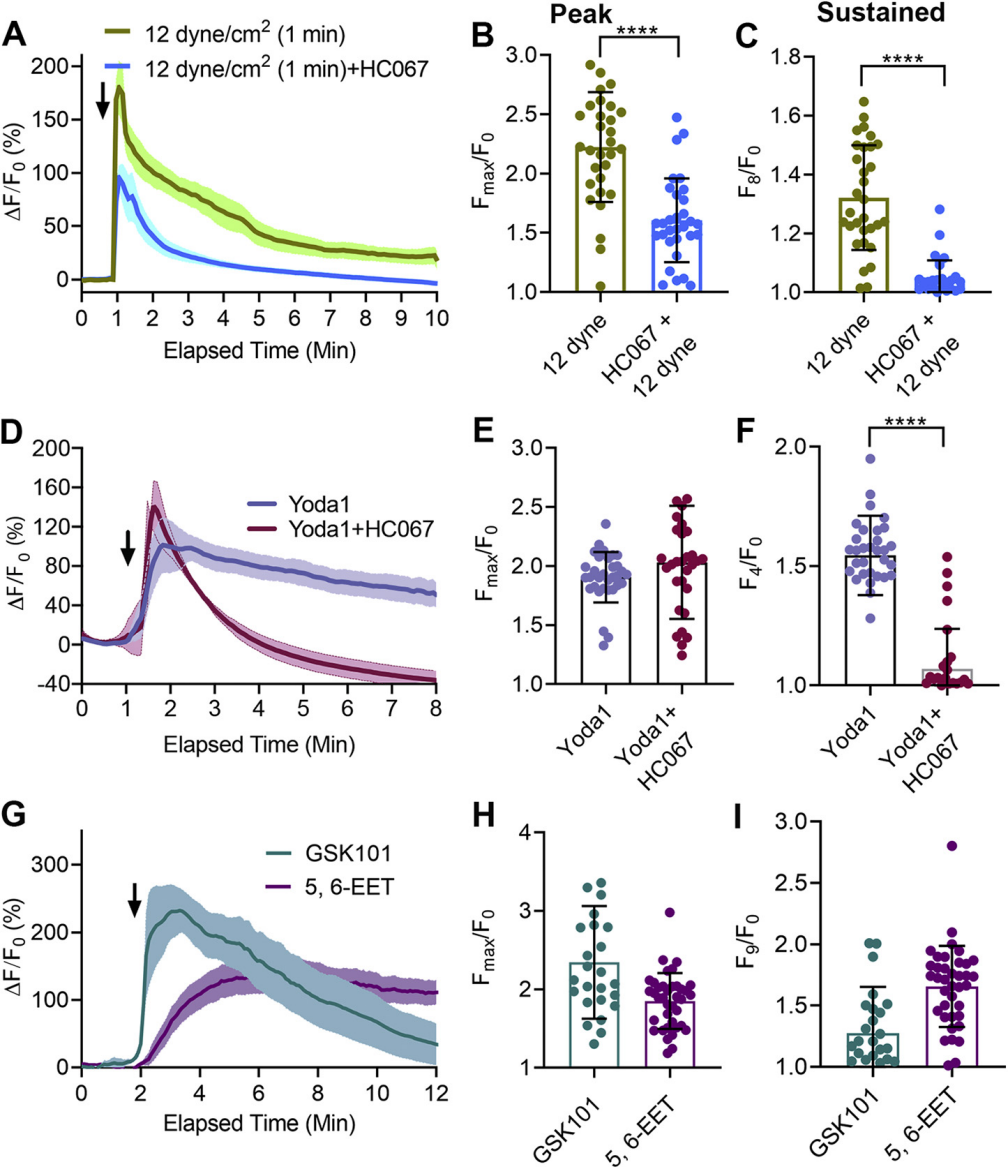
**TRPV4 antagonist, HC067, inhibited the Piezo1-induced sustained [Ca**^**2+**^**]**_**i**_**elevation in HUVECs.***A*–*C*, the effects of HC067 on fluid shear stress–induced [Ca^2+^]_i_ elevation. HC067 was applied 2 min before shear stress. *A*, the relative fluorescence intensity (ΔF/F_0_) of calcium dye over time with shear stress a 12 dyne/cm^2^ for 1 min with or without HC067 (1 μM). *B*, the average maximum peak [Ca^2+^]_i_ intensity and (*C*) the average peak [Ca^2+^]_i_ intensity calculated at 8 min after the force from 31 cells. *D*–*F*, the effect of HC067 (1 μM) on Yoda1 (5 μM)-induced [Ca^2+^]_i_ elevation. *D*, relative fluorescence intensity (ΔF/F_0_) of calcium dye over time. *E*–*F*, average maximum peak intensity and average peak intensity at 4 min from 29 cells. *G*–*I*, GSK101 (50 nM) and 5′,6′-EET (5 μM) increased [Ca^2+^]_i_. *G*, the relative fluorescence intensity of calcium dye, (*H*) average maximum peak [Ca^2+^]_i_ intensity, and (*I*) the average [Ca^2+^]_i_ intensity peak calculated at 9 min after initiation of force from 25 to 38 cells. Statistical analyses were performed using two-tailed Student’s *t* test, ∗∗∗∗*p* ≤ 0.0001. Data are shown as mean ± SD. 5′,6′-EET, 5′,6′-epoxyeicosatrienoic acid; HUVEC, human umbilical vein endothelial cell.

Although Piezo1 directly senses mechanical force (18), its fast inactivation kinetics and low single-channel conductance (15) render it unlikely to be directly responsible for the sustained elevation in [Ca^2+^]_i_ seen with higher shear forces. It is notable that endothelial cells also express another calcium-permeable ion channel—TRPV4 (4, 35). Even though cells expressing TRPV4 exhibit mechanosensitivity, direct channel activation by mechanical force has not been demonstrated (9, 27, 28). Therefore, the ability of shear stress to activate TRPV4 channels seems to be indirect. To evaluate the contribution of TRPV4 to sense shear force, we tested 1 μM HC067047 (HC067), a concentration that has been reported to completely block TRPV4 channel activity (21). HC067 slightly reduced the initial rise in [Ca^2+^]_i_ produced by shear force applied at 12 dyne/cm^2^ for 1 min but completely blocked the sustained calcium elevation (Fig. 2, *A*–*C*). This observation demonstrates that activation of the TRPV4 channel is responsible for the secondary, sustained elevation in [Ca^2+^]_i_ produced by shear force. Addtionally, HC067 (1 μM) completely blocked the Yoda1-induced sustained elevation in [Ca^2+^]_i_ without affecting the initial rise in [Ca^2+^]_i_, confirming that the Yoda1-induced and shear stress–induced secondary [Ca^2+^]_i_ phase is due to activation of TRPV4 and the initial transient [Ca^2+^]_i_ rise occurs through activation of Piezo1 (Fig. 2, *D*–*F*). To determine if selective activation of TRPV4 channels can reproduce the sustained [Ca^2+^]_i_ elevation, we tested the effects of the TRPV4 agonist, GSK1016790A (GSK101) and the endogenous TRPV4 agonist 5',6'-EET. Both GSK101 (50 nM) and 5',6'-EET (5 μM) produced sustained [Ca^2+^]_i_ elevations in HUVECs (Fig. 2, *G*–*I*); however, the kinetics of the [Ca^2+^]_i_ elevations were somewhat different. GSK101 caused a more rapid rise and subsequent decline in [Ca^2+^]_i_ rise than 5',6'-EET. A detailed study of the gating mechanisms, binding affinity, and half-lives of GSK101 and 5',6'-EET and their dose-dependent effects on TRPV4 activation will be needed to fully explain these changes. Along these lines, we observed that a lower concentration of 5',6'-EET reduced both the maximum and sustained [Ca^2+^]_i_ elevations in HUVECs (Fig. S2, *A*–*C*).

**Figure 3:**
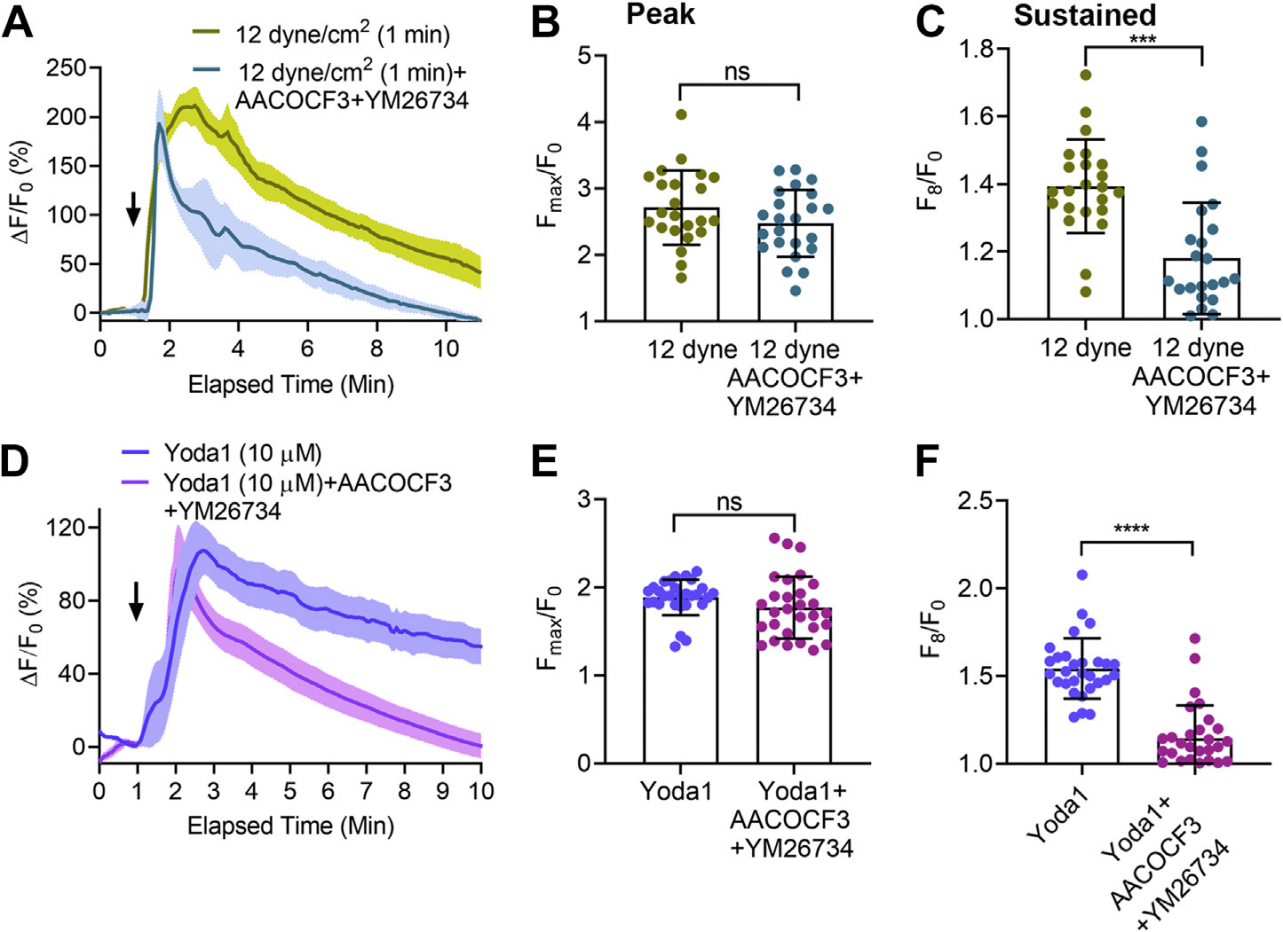
**PLA2 blockers inhibited the sustained [Ca**^**2+**^**]**_**i**_**elevation caused by fluid shear stress and Piezo1 agonist, Yoda1, in HUVECs.***A*–*F*, the effects of PLA2 blockers, AACOCF3 (30 μM) and YM26764 (10 μM), on Yoda1 (10 μM)-induced and shear stress (12 dyne/cm^2^ for 1 min)–induced [Ca^2+^]_i_ rise. *A* and *D*, the relative fluorescence intensity (ΔF/F_0_) of calcium dye over time with shear stress and Yoda1, respectively, with or without PLA2 blockers. *B* and *E*, the average maximum peak [Ca^2+^]_i_ intensity and (*C* and *F*) the average sustained [Ca^2+^]_i_ intensity calculated at 8 min after the stimuli from 31 cells for Yoda1 and 23 cells for shear stress. *Black arrows* show the time stimuli were applied. Statistical analyses were performed using two-tailed Student’s *t* test, ∗∗∗∗*p* ≤ 0.0001. Data are shown as mean ± SD. HUVEC, human umbilical vein endothelial cell.

Intracellular signals including PLA2 have been shown to activate TRPV4 (6), and we and others have previously reported that shear stress can increase PLA2 activity (33, 40). Therefore, to determine the intracellular process that is responsible for the effects of Piezo1 on TRPV4 channel opening, we applied PLA2 blockers and measured the effects of high shear stress and the selective Piezo1 agonist, Yoda1, on [Ca^2+^]_i_ in HUVECs. Exposure of HUVECs to Yoda1 throughout the period of imaging or high shear stress (12 dyne/cm^2^) for 1 min induced a prolonged increase in [Ca^2+^]_i_ (Fig. 3), raising the possibility that Piezo1 activation is coupled to TRPV4 channel opening. Treatment with the cytoplasmic PLA2 blocker AACOCF3 (30 μM) (41) and secretory PLA2 blocker YM26734 (10 μM) (42) significantly inhibited the Yoda1-induced and shear stress–induced sustained elevations in [Ca^2+^]_i_ (Fig. 3), indicating that Piezo1 regulates PLA2 activity, which is responsible for the subsequent activation of TRPV4.

Additionally, we demonstrated that the TRPV4 antagonist, HC067, blocked the 5',6'-EET–mediated [Ca^2+^]_i_ elevation in HUVECs, confirming that 5',6'-EET is the endogenous activator of the TRPV4 channel (Fig. S2, *D*–*F*). Consistent with the idea that TRPV4 is activated by PLA2-generated signaling molecules, we found that the cocktail of PLA2 blockers AACOCF3 (30 μM) and YM26734 (10 μM) did not affect the 5',6'-EET–stimulated and GSK101-stimulated elevation in [Ca^2+^]_i_ (Fig. S2, *G*–*J*).

### Piezo1 is required for shear stress–induced TRPV4 activation in HEK293T cells

**Figure 4:**
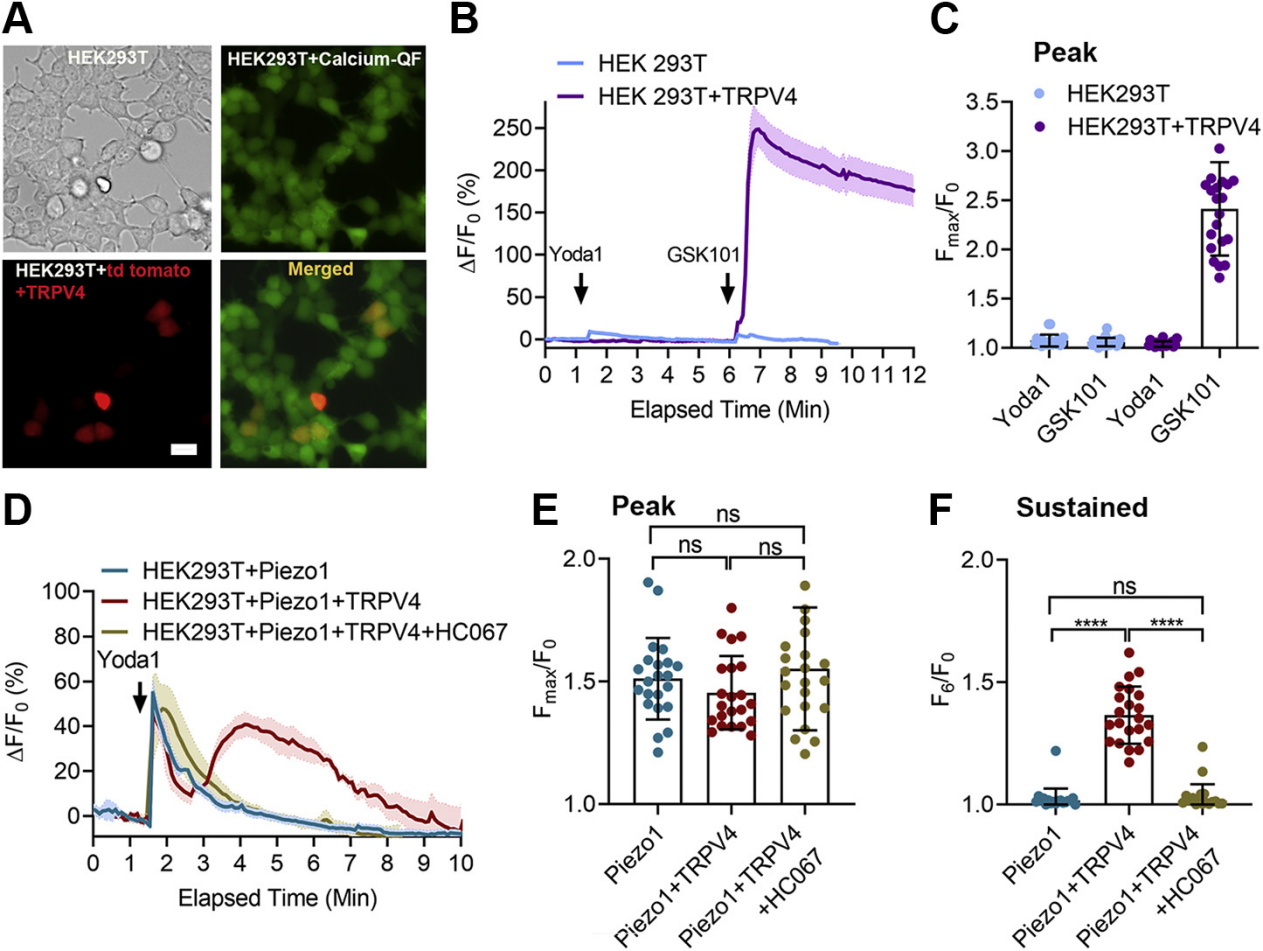
**TRPV4 mediated the sustained [Ca**^**2+**^**]**_**i**_**elevation in HEK293 T cells expressing Piezo1 and TRPV4 upon Yoda1 application.***A*, bright field and fluorescent images of HEK293T cells loaded with calcium dye, calcium-QF (*green*), cells cotransfected with plasmids containing tdTomato and TRPV4 cDNA (*red*), cotransfected cells merged with calcium-QF loaded cells (*yellow*). *B*, the relative fluorescence intensity (ΔF/F_0_) of calcium dye over time in nontransfected HEK293T cells and cells expressing TRPV4 with Yoda1 (5 μM) and GSK101 (100 nM). *Black arrows* show the time when Yoda1 and GSK101 were applied. *C*, the average maximum peak [Ca^2+^]_i_ intensity is shown for data in panel *B* from 21 cells. *D*, the relative fluorescence intensity (ΔF/F_0_) of calcium dye over time in HEK293T cells expressing Piezo1, Piezo1+TRPV4 with Yoda1 (5 μM), and Piezo1+TRPV4 with Yoda1 (5 μM) + HC067 (1 μM). *E*, the average maximum peak [Ca^2+^]_i_ intensity and (*F*) average sustained [Ca^2+^]_i_ intensity calculated at 6 min after the stimuli from 22 cells. Scale bar: 20 μm. Statistical analyses were performed using by 1-way ANOVA with Tukey’s multiple comparisons, ∗∗∗∗*p* ≤ 0.0001. Data are shown as mean ± SD.

**Figure 5:**
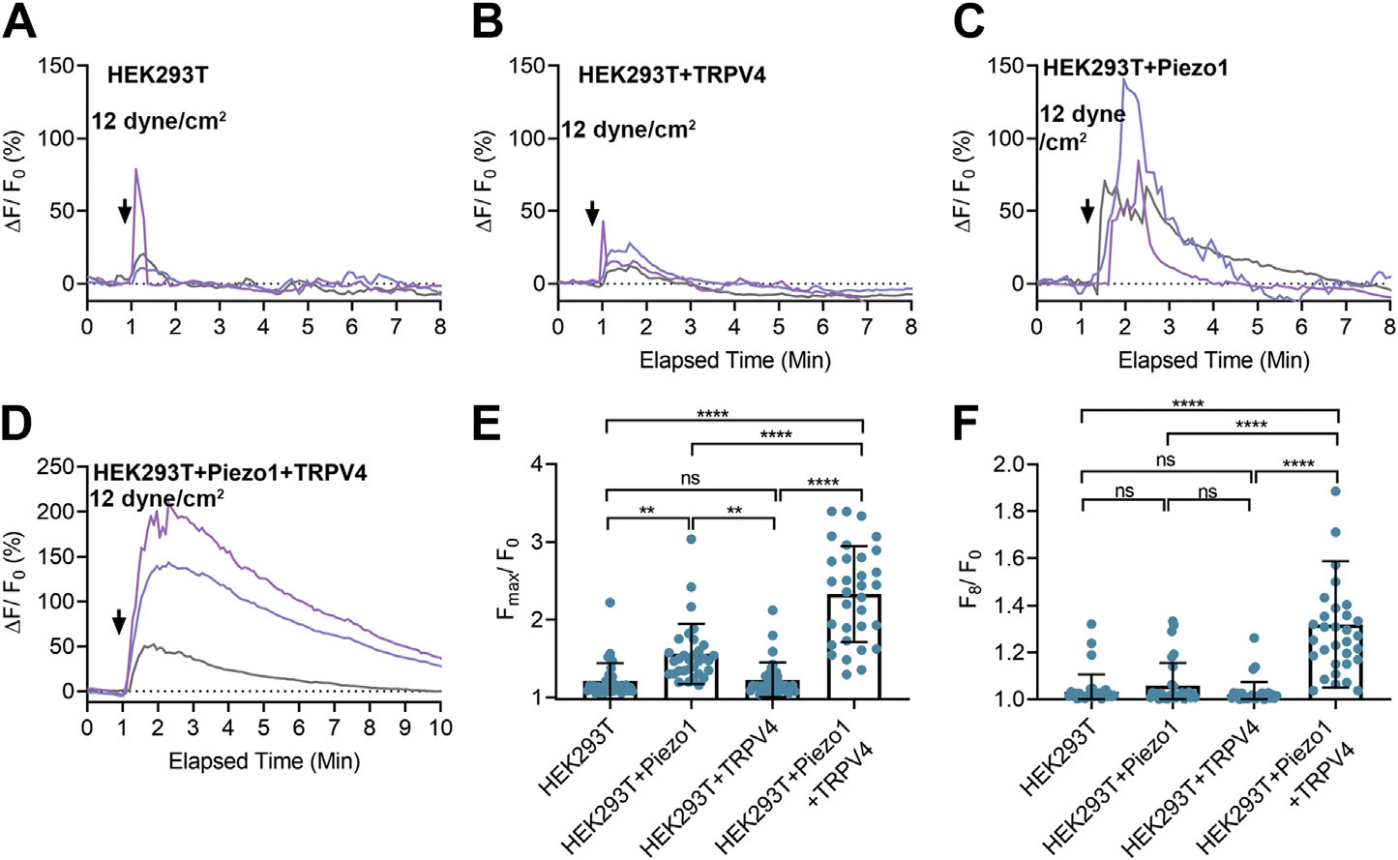
**High shear stress–mediated Piezo1 activation triggered TRPV4 channel opening.***A*–*D*, the relative fluorescence intensity (ΔF/F_0_) of calcium dye over time in nontransfected HEK293T cells and HEK293T cells expressing TRPV4, Piezo1, or Piezo1+TRPV4 upon application of shear stress (12 dyne/cm^2^ for 1 min). Each group represents [Ca^2+^]_i_ elevation in three individual cells. *Black arrows* show the time when Yoda1 was applied. *E*, the average maximum peak [Ca^2+^]_i_ intensity and (*F*) the average sustained [Ca^2+^]_i_ intensity calculated at 8 min after the stimuli from 31 cells. Statistical analyses were performed using by 1-way ANOVA with Tukey’s multiple comparisons, ∗∗*p* ≤ 0.01 ∗∗∗∗*p* ≤ 0.0001. Data are shown as mean ± SD.

Having demonstrated that TRPV4 is responsible for the Piezo1-induced sustained [Ca^2+^]_i_ elevation in HUVECs, we next established a cell system in which the effects of Piezo1 and TRPV4 could be evaluated independently. Here we used HEK293T cells in which endogenous *Piezo1* was deleted (43). We then induced transient expression of *Piezo1*, *TRPV4*, and *Piezo1+TRPV4*. Except for nontransfected cells, tdTomato cDNA was cotransfected with other genes to mark the positively transfected cells for calcium imaging. We observed that ∼98% of tdTomato-positive cells responded to the respective ion channel agonists, indicating high cotransfection efficiency. As expected, nontransfected cells did not respond to Yoda1 or shear stress (12 dyne/cm^2^ for 1 min), and cells expressing TRPV4 responded to GSK101 (100 nM), but not to Yoda1 (5 μM) (Fig. 4, *A*–*C*). In cells expressing Piezo1, Yoda1 produced a significant but transient [Ca^2+^]_i_ elevation. However, in cells expressing both Piezo1 and TRPV4, Yoda1 produced brisk and sustained [Ca^2+^]_i_ elevations; the latter was completely blocked by the TRPV4 antagonist HC067 (Fig. 4, *D*–*F*). Shear stress produced effects similar to Yoda1 on HEK293T cells expressing *Piezo1*, *TRPV4*, and *Piezo1+TRPV4* (Fig. 5). These results confirm that the initial transient increase in [Ca^2+^]_i_ induced by Yoda1 and shear stress is caused by activation of Piezo1 and the secondary sustained phase of [Ca^2+^]_i_ elevation resulted from activation of TRPV4. In approximately 10% of nontransfected and TRPV4-expressing cells, shear stress produced a transient (30 s) spike in [Ca^2+^]_i_, raising the possibility that other mechanically sensitive ion channels may exist in HEK293T cells.

### Piezo1-induced AJ disruption is the consequence of TRPV4 activation

**Figure 6:**
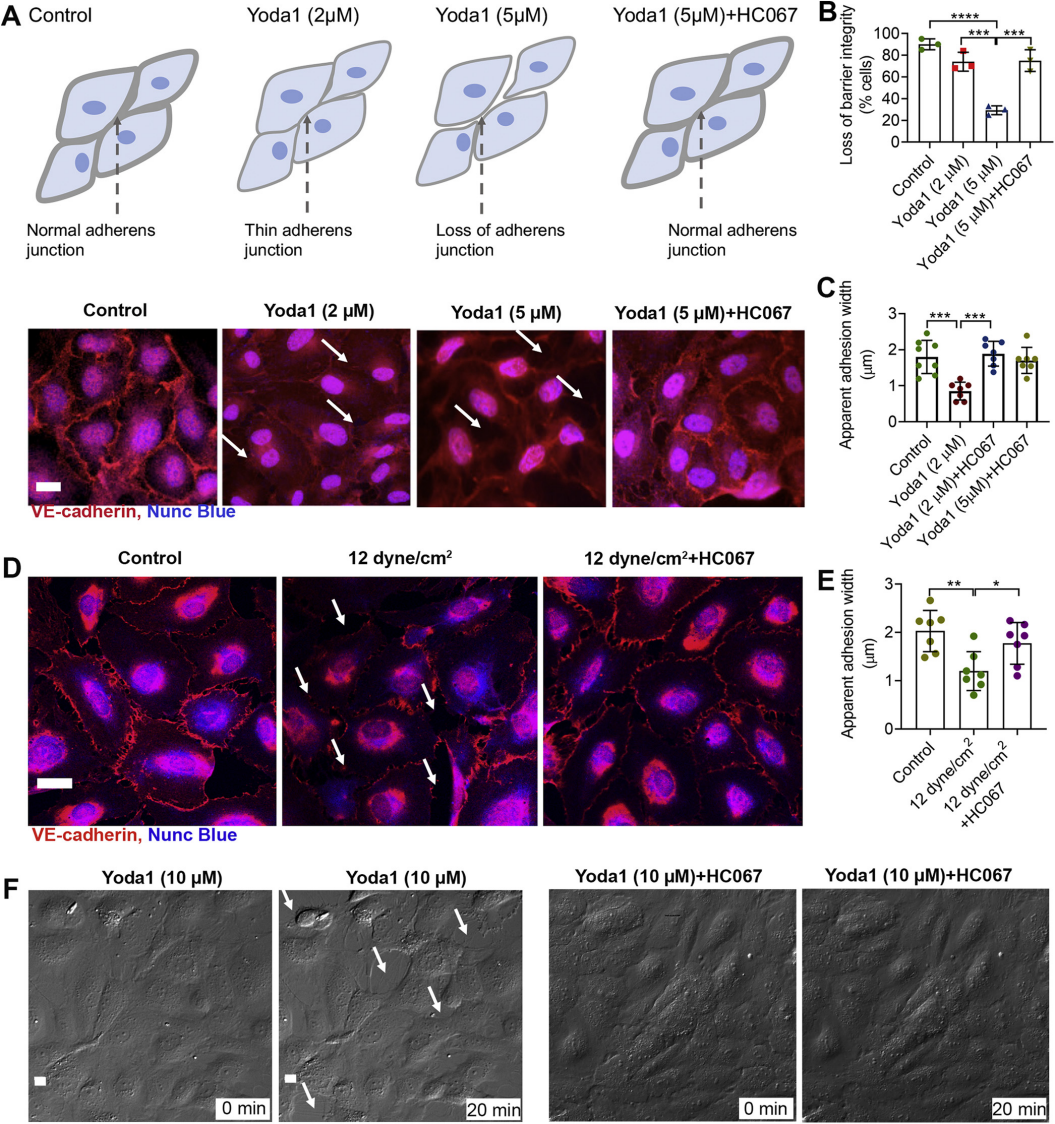
**TRPV4 antagonist, HC067, prevented Piezo1-mediated loss of AJs in HUVECs.***A*–*D*, accumulation of the AJ protein, VE-cadherin, at junctions of HUVECs in monolayer cultures is shown by *red* immunostaining along the cell membrane of endothelial cells. *A*, the images and cartoons show that application of Yoda1 for 30 min caused thinning (2 μM) or loss (5 μM) of VE-cadherin in endothelial cells. HC067 (1 μM) prevented Yoda1-induced loss of VE-cadherin. *B*, the number of cells with loss of cellular integrity was calculated from three experiments. *C*, the width of VE-cadherin (*red*) staining was quantified from data shown in *A*. *D*, effects of laminar fluid shear stress (12 dyne/cm^2^ for 10 min) with or without HC067 on VE-cadherin (*red*) accumulation at junctions of HUVEC monolayers are shown. *E*, quantification of the apparent width of VE-cadherin (*red*) was calculated from data shown in *D*. *F*, live cell DIC images of HUVECs in monolayer culture before or 20 min after Yoda1 (10 μM) or Yoda1 + HC067 (5 μM) show the loss of cellular junctions over time, retraction of cells (a cell boundary marked in *white dotted lines* before and after Yoda1), and formation of intracellular gaps (indicated by *white arrows*). Data are shown as mean ± SD. Statistical analyses were performed using two-tailed Student’s *t* test; multiple groups were analyzed by 1-way ANOVA with Tukey’s multiple comparisons. ∗*p* ≤ 0.05; ∗∗*p* ≤ 0.01, ∗∗∗*p* ≤ 0.001; ∗∗∗∗*p* ≤ 0.0001. Scale bar: 10 μm. AJ, adherens junction; HUVEC, human umbilical vein endothelial cell.

AJs consist of α-catenin, β-catenin, and p120-catenin and the transmembrane adhesive protein VE-cadherin (11, 44). Activation of Piezo1 disrupts vascular AJs and increases vascular permeability (11). To determine if this disruption occurs through Piezo1 on vascular endothelial cells, we treated monolayer HUVEC cells with Yoda1 (2 μM) for 30 min and observed a reduction in VE-cadherin expression at AJs (Fig. 6*A*). The overall apparent width of VE-cadherin at AJs was decreased significantly with Yoda1 (2 μM) (Fig. 6*C*). A higher concentration of Yoda1 (5 μM) decreased the accumulation of VE-cadherin and disrupted HUVEC integrity (Fig. 6, *A*–*B*). We proposed that these changes were due to Piezo1-triggered TRPV4 activation, causing secondary calcium overload. To determine if the effect of Yoda1 on VE-cadherins was due to activation of TRPV4, we evaluated the action of HC067 (1 μM). Treatment with HC067 stabilized the VE-cadherin at AJs and also protected the integrity of HUVECs in monolayer culture (Fig. 6, *A*–*B*). These findings indicate that Yoda1-induced loss of AJs is through the combined actions of Piezo1 and TRPV4. We observed that a high concentration of Yoda1 (10 μM) caused cell retraction and produced paracellular gaps in monolayer cultures (Fig. 6*F* and Movie S1). Paracellular gaps were the consequence of the reduction in the cell surface area. Cellular disruption due to Yoda1 was absent when the TRPV4 blocker HC067 was added to the media (Fig. 6*F* and Movie S1).

Subjecting HUVEC monolayers to 12 dyne/cm^2^ for 10 min reduced VE-cadherin at AJs and decreased the apparent width of individual cells. Knowing that through Piezo1, shear stress triggers TRPV4 activation which is responsible for producing high levels of [Ca^2+^]_i_, we postulated that blocking TRPV4 would protect against the loss of VE-cadherin caused by Yoda1. Thus, if TRPV4 was responsible for the sustained [Ca^2+^]_i_ elevation that causes the loss of VE-cadherin, then blocking TRPV4 would be expected to protect against disruption of AJs. We observed that HC067 blocked shear stress–mediated loss of VE-cadherin at AJs (Fig. 6*D*) and preserved the apparent width of VE-cadherin (Fig. 6*E*).

### Inhibition of Piezo1 channels protected against shear stress–induced actin disorganization

**Figure 7:**
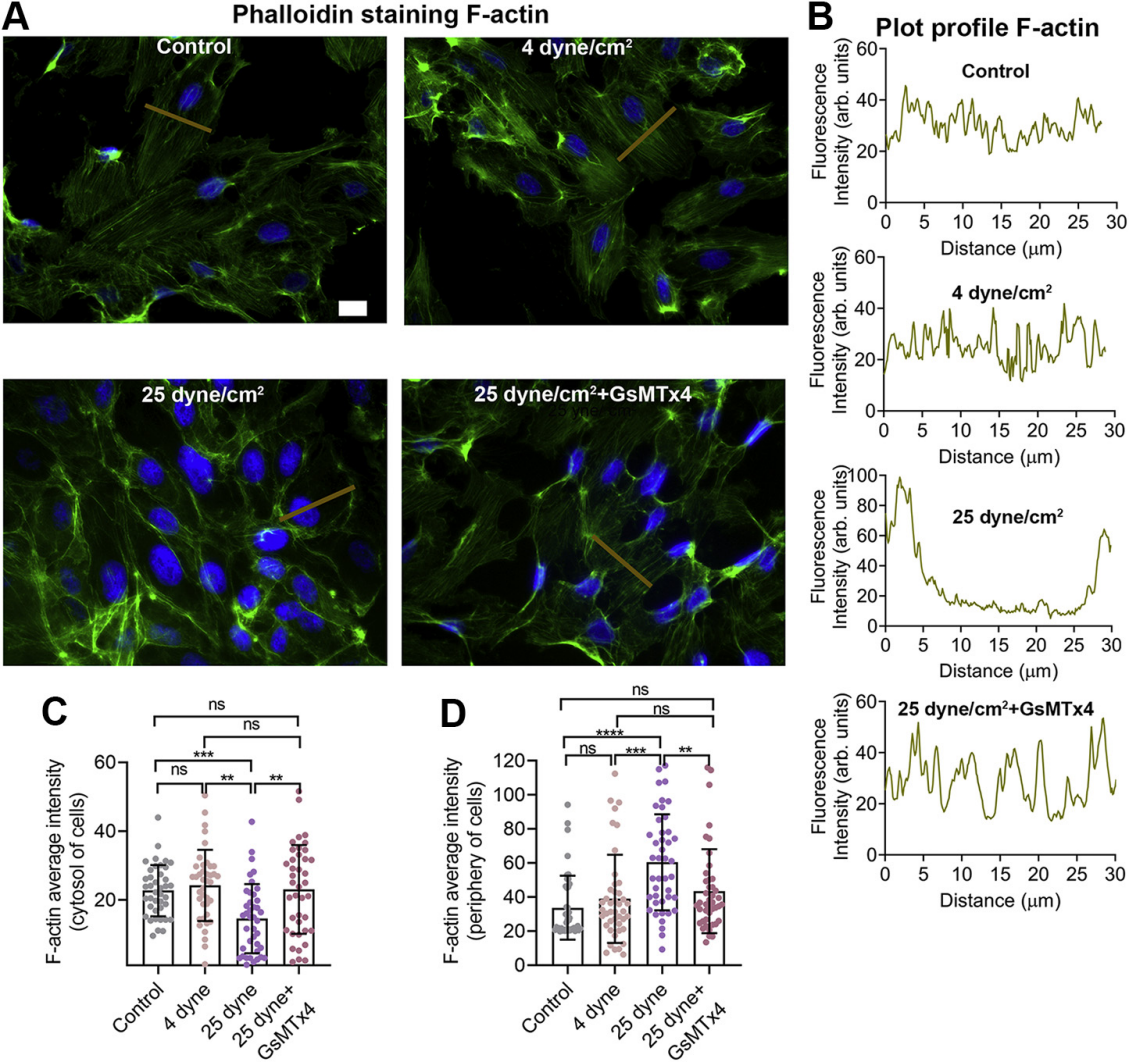
**GsMTx4 inhibited F-actin disorganization in HUVECs caused by high shear stress.***A*, representative images show F-actin filament organization (*green*) and distribution in HUVECs under culture conditions of no shear stress (control panel), or shear stress applied for 10 min at 4 dyne/cm^2^ and 25 dyne/cm^2^ with or without GsMTx4 (10 μM). Data are representative of three experiments. *B*, plot profile of F-actin from a representative cell. As represented in *A*, a line (*tan color*) was drawn perpendicular to F-actin orientation across which intensity profiles for Alexa 488 phalloidin were measured. *C*–*D*, the average intensities of F-actin were measured at the cytosolic and peripheral regions from 40 cells. Data are shown as mean ± SD. Statistical analyses were performed using 1-way ANOVA with Tukey’s multiple comparisons. ∗∗*p* ≤ 0.01, ∗∗∗*p* ≤ 0.001; ∗∗∗∗*p* ≤ 0.0001. Scale bar: 10 μm. HUVEC, human umbilical vein endothelial cell.

Cytoskeleton remodeling in response to the physiological levels of shear stress is necessary for endothelial cell migration and maintaining vascular integrity (1, 45). However, shear stress generated under conditions of high vascular pressure or turbulence has adverse effects on the cytoskeleton which can lead to increased endothelial cell stiffness, vascular permeability, and transmigration of leukocytes (1, 11, 46, 47). We analyzed F-actin fiber distribution using F-actin plot profiling and measured the F-actin orientation and intensity at cytosolic and peripheral regions of the cell (Fig. 7, A–*D*). We observed that high shear stress (25 dyne/cm^2^ for 10 min) disrupted the orientation of F-actin in HUVECs in contrast to lower shear force (4 dyne/cm^2^ for 10 min) (Fig. 7, *A*–*B*). High shear stress triggered the formation of F-actin bundles indicative of fiber polymerization (Fig. 7, *A*–*B*). The F-actin intensity frequency distribution predicted that high shear stress caused F-actin fibers to translocate from the perinuclear region of the cell to the periphery where a high density of uneven clusters appeared (Fig. 7, *A*–*D*). The uniform thickness of F-actin fibers generally observed under low shear stress conditions was lost and extensive polymerization of thick F-actin bundles appeared at the periphery of cells subjected to high shear stress (Fig. 7, *A*–*D*). These effects of high shear forces were prevented in cells treated with GsMTx4, confirming that high shear stress–mediated F-actin disorientation required Piezo1 channel activation (Fig. 7).

### Piezo1-mediated F-actin disruption depends on TRPV4 activation

**Figure 8:**
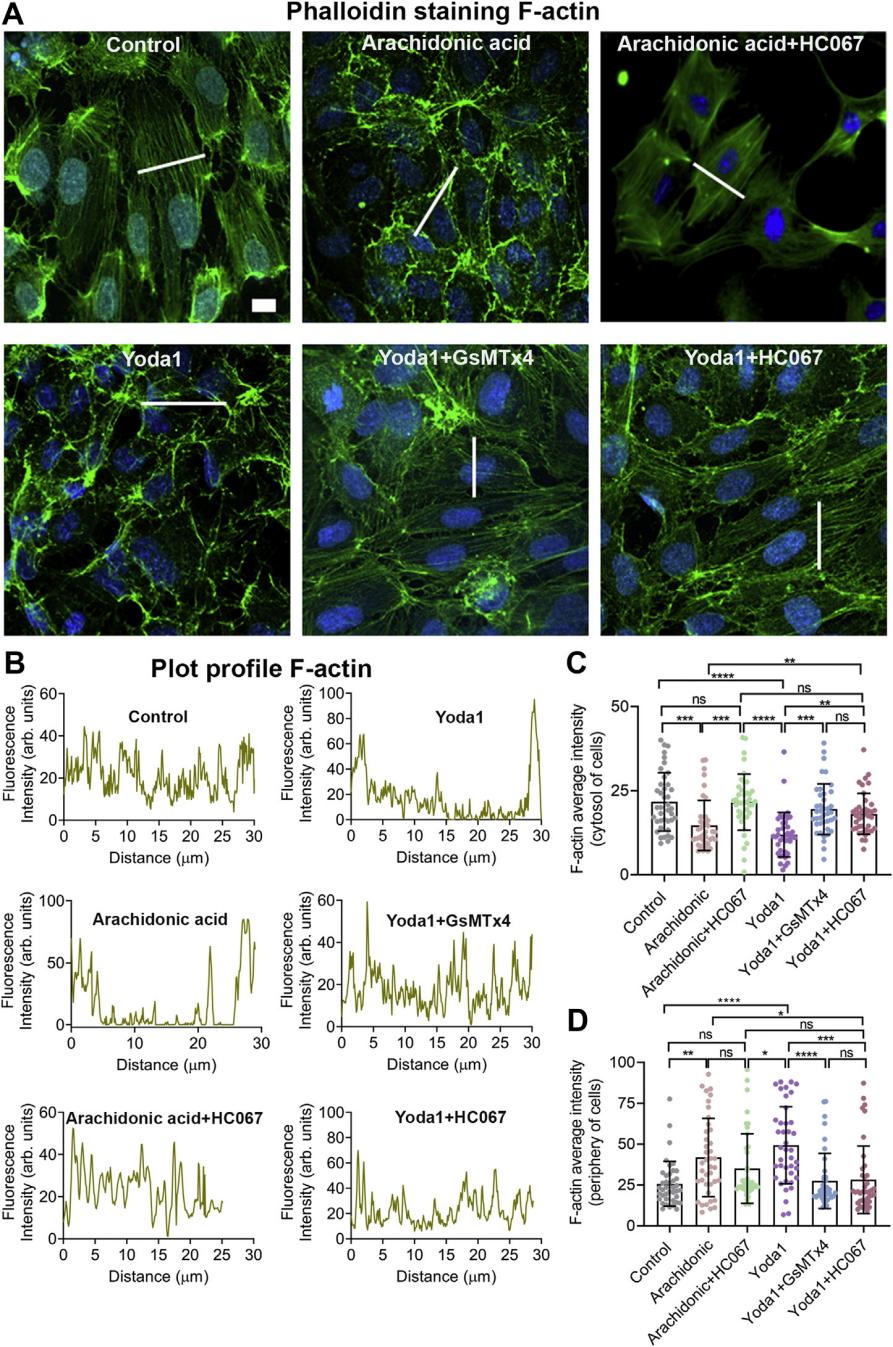
**Piezo1 antagonist, GsMTx4, and TRPV4 antagonist, HC067, protected against Yoda1-induced F-actin disruption in HUVECs.***A*, representative images of HUVECs show the distribution of F-actin filaments (Alexa 488 phalloidin, *green*) 10 min after treatment with DMSO (control), Yoda1 (10 μM), Yoda1 (10 μM) + GsMTx4 (10 μM), and Yoda1 (10 μM) + HC067 (1 μM). Images are representative of three experiments. *B*, plot profile of F-actin from a representative cell. A *white line* was drawn perpendicular to filament orientation for plot profile analysis of F-actin, and intensity profiles of Alexa 488 phalloidin across these lines were measured. *C*–*D*, the average intensities of F-actin were measured at cytosolic and peripheral regions from 40 cells. Data are shown as mean ± SD. Statistical analyses were performed using 1-way ANOVA with Tukey’s multiple comparisons. ∗*p* ≤ 0.05; ∗∗*p* ≤ 0.01, ∗∗∗*p* ≤ 0.001; ∗∗∗∗*p* ≤ 0.0001. Scale bar: 10 μm. HUVEC, human umbilical vein endothelial cell.

To determine if activation of Piezo1 can affect the F-actin disorganization and might be the mechanism through which shear stress causes cytoskeletal remodeling, we applied the Piezo1 agonist, Yoda1 (10 μM), for 10 min to HUVECs. Without Yoda1 administration (control), the F-actin fibers were parallel in orientation and evenly distributed. Application of Yoda1 (10 μM) caused a drastic change in HUVEC morphology with apparent cell contraction and reduction in cell surface area, complete loss of F-actin fiber distribution, and accumulation of high-density clusters of F-actin fibers at the periphery of cells and loss of F-actin fibers in the cytosolic region of the cells (Fig. 8). These Yoda1-induced changes were significantly reduced by the TRPV4 blocker, HC067 (1 μM), confirming that Piezo1-mediated F-actin disorganization required TRPV4 activation (Fig. 8). We determined that arachidonic acid (10 μM), an endogenous mediator of TRPV4 activation, triggered the same pattern of F-actin disorganization and gathering of high-density clusters of F-actin fibers in the periphery of HUVECs, all of which were inhibited by HC067 (Fig. 8). These findings signify that TRPV4 activation can cause cytoskeleton damage and that shear stress–triggered remodeling of F-actin and disruption of F-actin fibers results from the combined actions of Piezo1 and TRPV4.

### Blockade of TRPV4 inhibits Piezo1-mediated monocyte adhesion to endothelial monolayers

**Figure 9:**
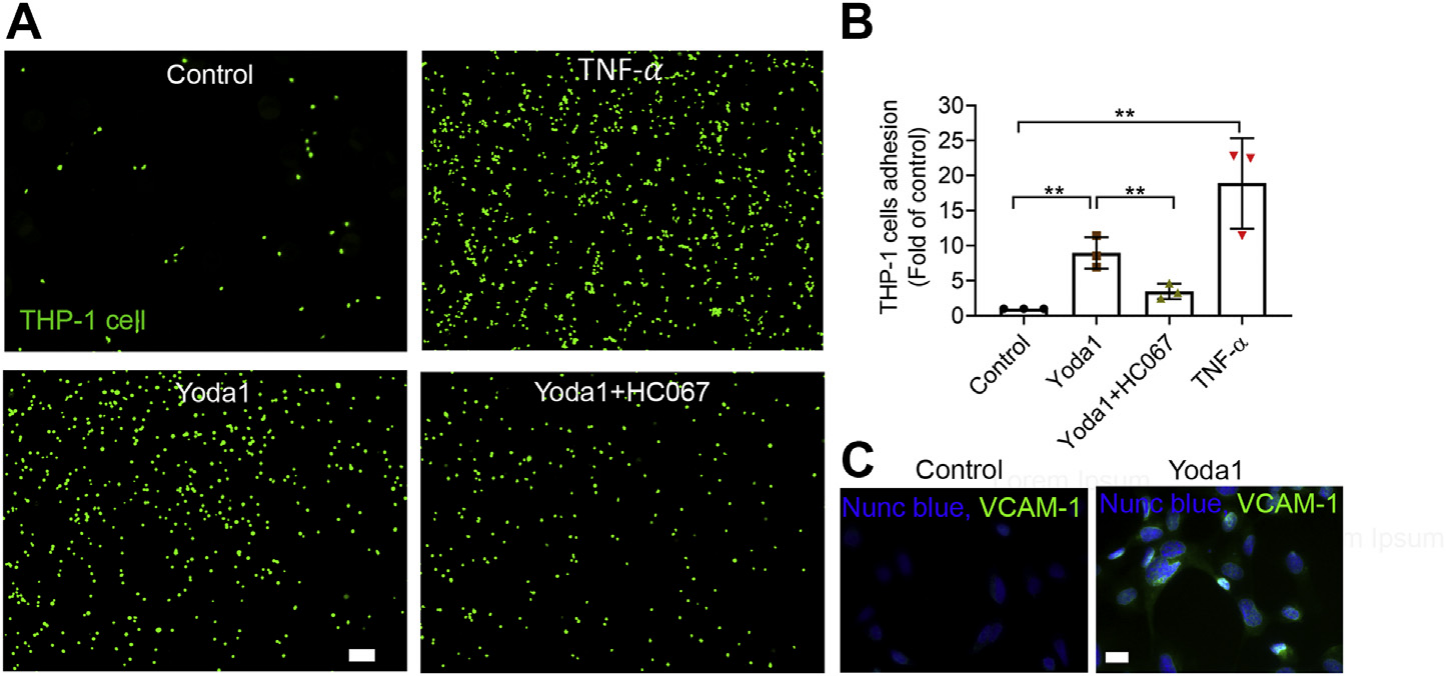
**TRPV4 antagonist, HC067, prevented Yoda1-mediated THP-1 cell adhesion to HUVEC monolayer cultures.***A*, representative images showing that HC067 (1 μM) prevented the Piezo1 agonist Yoda1 (1 μM)-mediated THP-1 cell (*green*) adhesion to HUVEC monolayers. HUVEC monolayers were incubated with DMSO (control), Yoda1, Yoda1+HC067, and TNF-α (10 ng/ml) as a positive control for 10 h, and then THP-1 cells were added for 1 h to attach. Scale bar: 100 μm. *B*, the quantification of data A from three independent experiments. *C*, the expression of VCAM1 (*green*) in endothelial cells with and without Yoda1 (1 μM) for 10 h. Nuclei were stained with Nunc Blue. Scale bar: 10 μm. Individual data points are shown with mean ± SD; statistical analyses were performed using by 1-way ANOVA with Tukey’s multiple comparisons. ∗∗*p* ≤ 0.01. HUVEC, human umbilical vein endothelial cell.

Disruption of normal cell-to-cell contacts in the vascular endothelium accompanies excessive shear force and leads to an inflammatory response characterized by monocyte adhesion (3, 36). Having demonstrated that shear force activates Piezo1 which can disrupt AJs and form paracellular gaps in confluent monolayers, we next evaluated the effects of Piezo1 on endothelial cell activation and adhesion protein expression, which are two of the early processes in atherosclerosis (48). Exposing endothelial cells to Yoda1 (1 μM) for 10 h increased expression of the adhesion protein VCAM1 and stimulated the attachment of monocytes (THP-1 cells) (Fig. 9). THP-1 attachment was prevented by the TRPV4 antagonist HC067 (Fig. 9, *A*–*B*). These findings indicate that the adverse effects of Piezo1-induced changes in endothelial cell adhesion led to the attraction of inflammatory cells and were mediated by TRPV4.

## Discussion

Calcium signaling in endothelial cells is a highly regulated process (36). Under physiological conditions, the amplitude and frequency of [Ca^2+^]_i_ oscillations in endothelial cells maintain cellular integrity, but stimulation by inflammatory mediators such as histamine, bradykinin, and thrombin increases intracellular calcium up to 10 fold (36). High sustained [Ca^2+^]_i_, leading to calcium overload, alters normal calcium signaling pathways and causes cytoskeletal disorganization and AJ disassembly. Together these processes facilitate transendothelial migration of leukocytes.

High blood pressure and shear stress also have been associated with endothelial dysfunction (1, 3, 13, 36, 49). High blood pressure– or shear stress–mediated calcium overload contributes to cytoskeletal disorganization, AJ disassembly, and leukocyte transendothelial migration (2, 10, 11, 17, 34, 47). However, the severity of damage is endothelium-dependent, as the sensitivity to shear stress differs between the arterial and venous systems (50). Endothelial cells sense pressure and shear forces through calcium-permeable mechanosensitive ion channels that convey extracelluar signals to maintain cellular integrity. The extent of calcium influx depends upon the strength of blood flow shear stress (51) and the number of mechanosensitive ion channels in the particular endothelium. We demonstrated in HUVECs that prolonged high shear stress produced a sustained elevation in [Ca^2+^]_i_ in contrast to low levels of shear stress or transient high shear stress. HUVECs are typical of other endothelial cells and express two types of mechanosensitive ion channels, Piezo1 and TRPV4, which have been implicated in vascular pathophysiological conditions (10, 11, 17, 52, 53). Either Piezo1 or TRPV4 may contribute to abnormally high [Ca^2+^]_i_ and may even initiate pathological events in endothelial cells (53, 54, 55).

Piezo1 has a low single-channel conductance of approximately 22 pS and is a fast inactivating channel (15). Therefore, it seemed unlikely that Piezo1 alone could produce the prolonged elevation in [Ca^2+^]_i_ to induce a calcium overload state necessary to disrupt endothelial cell integrity. This led us to search for another calcium signaling pathway or calcium-permeable ion channel that might be involved. TRPV4 was a likely candidate due to its slow inactivation kinetics and high single-channel conductance of approximately 60 pS (56). However, unlike Piezo1, TRPV4 does not appear to possess direct mechanosensing properties (7, 27, 28, 29, 40). Although under certain experimental conditions, TRPV4 has been shown to become activated by physical forces, such as shear stress, osmotic pressure, and mechanical stretching (4, 23, 26, 27, 57, 58), the mechanical gating of TRPV4 has not been described. In whole-cell systems such as *Xenopus* oocytes transfected with TRPV4 and TRPV4-expressing endothelial cells, hypotonicity and mechanical forces increased TRPV4 activity (4, 34, 58). However, in cell-free systems (*e.g.*, inside-out membrane patches or lipid bilayers), similar responses have not been studied. In analogous experiments, in cells, TRPV4 was sensitive to changes in temperature, but the same sensitivity was not seen in cell-detached, inside-out patches (56, 59). These findings suggest that mechanosensing and temperature sensing may not be inherent properties of TRPV4 itself.

The mechanosensitive nature of the TRPV4 channel has been controversial. Urothelial cells that express endogenous TPPV4 and *Xenopus* oocytes transfected with TRPV4, but not TRPV4-expressing HEK293 T cells, were activated by membrane stretch (26, 27, 31, 58). In the absence of confounding factors, if TRPV4 directly senses mechanical force, one would expect all TRPV4-expressing cells to respond to mechanical stimuli. Our observation that deletion of Piezo1 in TRPV4-expressing HEK293 T cells eliminated their ability to respond to shear force supports the concept that Piezo1 but not TRPV4 is mechanosensitive and that TRPV4 activation is downstream to Piezo1 activation. It is likely that the level of Piezo1 expression and the number of Piezo1 channel openings affect the extent of the TRPV4 channel opening and may explain, at least in part, the heterogeneous response of TRPV4-expressing cells to mechanical forces.

During shear stress, TRPV4 is activated by 5',6'-EET, an endogenous ligand derived from arachidonic acid through the activation of PLA2 (28, 33). Recently, we discovered that high pressure activated Piezo1, PLA2, and TRPV4 in pancreatic acinar cells, producing a sustained [Ca^2+^]_i_ elevation. However, in acinar cells from TRPV4-knockout mice, high pressure caused only a transient elevation in [Ca^2+^]_i_ (33). HUVECs sense mechanical force in a similar manner. In HUVECs, shear stress stimulates TRPV4 channel opening through Piezo1-stimulated activation of PLA2. A cocktail of PLA2 blockers (YM26734 and AACOCF3) significantly inhibited the sustained [Ca^2+^]_i_ elevation produced with Yoda1 and shear stress, while the TRPV4 antagonist HC067 completely blocked the sustained calcium influx, suggesting other signaling pathways might be operating along with PLA2 to activate TRPV4. Binding of calcium ions to PLA2 accelerates enzyme activity, which initiates 5',6'-EET production and TRPV4 channel activation. It appears that a threshold level of peak [Ca^2+^]_i_ was necessary to induce PLA2 activation. It is likely that peak [Ca^2+^]_i_ is controlled by the number of Piezo1 channel openings. It could be that brief shear stress activated only a subset of Piezo1 channels, resulting in submaximal [Ca^2+^]_i_ elevation which was insufficient to activate PLA2 in endothelial cells. In contrast, Yoda1, which would be expected to activate most Piezo1 channels, mimicked the effects of prolonged high shear stress–mediated [Ca^2+^]_i_ elevation. Like HUVECs, Yoda1 and high shear stress caused sustained [Ca^2+^]_i_ elevation in HEK293T cells expressing both Piezo1 and TRPV4. Cells expressing TRPV4 alone were nonresponsive to Yoda1 and high shear stress, and cells expressing only Piezo1 exhibited only transient calcium elevations, confirming that Piezo1 and TRPV4 coupling during high mechanical force is a generalized process.

Basal endothelial calcium levels control vascular permeability and contraction. Notably, basal calcium levels are altered in hypertension and high shear stress, which leads to disruption of the cell–cell junctional protein VE-cadherin and loss of endothelial barrier function. Impaired endothelial barrier function contributes to atherosclerosis which is a major cause of mortality among patients with hypertension. It was recently reported that blocking Piezo1 or TRPV4 prevented hypertension-associated vascular hyperpermeability, but the underlying mechanisms or possible interactions were not uncovered (10, 11). Nevertheless, our findings demonstrate how TRPV4 may contribute to microendothelial permeability (10, 34).

It has been shown that TRPV4-knockout mice are protected from lung vascular hyperpermeability in response to high pulmonary venous pressure (PVP) (20, 57, 60, 61). Similarly, pharmacological blockade with the TRPV4 antagonist GSK2193874 also protected against PVP-induced permeability of the lung (10). It was recently reported that Piezo1 senses high vascular pressures at the lung endothelial surface and is responsible for vascular hyperpermeability and pulmonary edema (11). Thus, it appears that the high PVP–mediated vascular hyperpermeability is due to both Piezo1 and TRPV4. Our results demonstrated that Piezo1 activation caused the loss of VE-cadherin at the cell–cell junction and disrupted endothelial barrier integrity which was prevented by TRPV4 blockade. Moreover, TRPV4 was responsible for Piezo1-initiated cell retraction and paracellular gap formation in monolayer cultures. Thus, the combined actions of both Piezo1 and TRPV4 may contribute to the endothelial vascular barrier dysfunction in hypertension. Shear stress also accompanies increased viscosity of blood which is a risk factor for deep vein thrombosis (23, 62, 63). It will be interesting to determine if targeting Piezo1 or TRPV4 channels can improve either of these clinical conditions.

Fluid shear stress caused by blood flow is a major determinant of vascular remodeling and arterial tone and can lead to the development of atherosclerosis (36, 47). High shear stress–mediated calcium signaling activates endothelial cells, cytoskeleton remodeling, and production of inflammatory cytokines and chemokines which facilitate leukocyte rolling, adhesion, and transendothelial migration (36). Our results suggest that all of these effects can be initiated by Piezo1 activation. Therefore, loss of endothelial barrier integrity and monocyte adhesion which are integral to atherosclerosis may be directly related to Piezo1 (19, 55). Although our findings provide insight into the relationship between Piezo1 activation and deleterious effects on endothelial cells, it is important to note that development of atherosclerosis is a chronic process and is best studied in animals. Therefore, the relationship between Piezo1 and TRPV4 in the endothelium and initiation and perpetuation of atherosclerosis is more complicated than what can be studied *in vitro*. Nevertheless, the effects of Piezo1 on endothelial cell integrity may be an early step in the disease.

Piezo1 initiated the remodeling in our *in vitro* system. It is possible that a similar mechanism occurs under conditions of elevated shear stress *in vivo*. Due to the rate of blood flow, the endothelium in the arterial system is exposed to higher shear stress forces than the venous endothelium. In addition, transient or pulsatile shear stress is beneficial in the arterial system (2). In our experiments, using cells of venous origin, high shear stress of 25 dyne/cm^2^ for minutes at a time caused adverse effects. Regional distribution of both Piezo1 and TRPV4 throughout the vascular endothelium has not been extensively evaluated. It is possible that deleterious effects of high pressure do not occur in some vascular beds due to the absence of coexpression of Piezo1 and TRPV4 in the same cell. Our findings suggest that cells lacking either Piezo1 or TRPV4 would be protected from high shear stress–induced changes in cytoskeleton, cell retraction, and inflammatory cell adhesion. Similarly, pharmacological blockade of either Piezo1 or TRPV4 would be expected to prevent the deleterious effects of shear stress in the endothelium. However, identification and functional chacterization of both Piezo1 and TRPV4 throughout the vascular endothelium would be needed before considering therapeutics for cardiovascular diseases directed against these ion channels.

The deleterious effects of shear stress in endothelial cells are secondary to sustained Ca^2+^ influx through TRPV4 channels. Therefore, the extent of Ca^2+^ influx could be influenced by several factors including the strength and duration of mechanical force, the level of PLA2 activity, and TRPV4 abundance. Our findings suggest that Piezo1 or TRPV4 alone is not sufficient to transduce mechanical force into pathological events. We have previously demonstrated that coupling of Piezo1 with TRPV4 is responsible for the effects of high pressure in pancreatic acinar cells (33). If functional coupling between Piezo1 and TRPV4 is a generalized process in which TRPV4 translates the mechanical force sensed by Piezo1 into pathological events, then other organ systems like urinary bladder and bone tissue where Piezo1 and TRPV4 are coexpressed (21, 64, 65, 66, 67) may also be amenable to targeting Piezo1 or TRPV4 to prevent adverse effects of pressure or shear stress.

## Experimental procedures

### Cell culture and expression of Piezo1 and TRPV4

Primary HUVECs (CC-2519) were obtained from Lonza. Cells were cultured with Endothelial Cell Growth Media (Cell Applications; 211–500) in a humidified incubator at 37 °C and 5% CO_2_ (68). Cells were fed every 2 days and were not allowed to grow to confluence unless required for a specific experiment. Cells were used for experiments between passage numbers 2 to 5. HUVECs were passaged with 0.025% Trypsin/EDTA solution (TE, #R001100, Thermo Fischer Scientific). THP-1 cells, a monocyte cell line, were obtained from ATCC (TIB-202) (69). The suspension culture of THP-1 was grown in RPMI 1640 + sodium pyruvate (1 mM) + Hepes (10 mM) + glucose (10 mM) + 10% FBS +0.05 mM beta-mercaptoethanol in a humidified incubator at 37 °C and 5% CO_2_. Like HUVECs, THP-1 cells were fed every 2 days, and during culture, the cell number was not allowed to exceed 1 × 10^6^. Primocin (InvivoGen) at a concentration of 100 μg/ml was added to cell culture media.

Piezo1-deleted HEK293 T cells were transiently transfected with pcDNA3.1 plasmids containing mouse *Piezo1* or human *TRPV4* (15, 22) alone or together using Lipofectamine 3000 (Invitrogen) according to the manufacturer’s protocol. A plasmid containing tdTomato was cotransfected with targeted cDNA at a 1:8 M ratio for the selection of positively transfected cells. Calcium imaging in the transfected cells was performed 36 to 48 h post transfection.

### Calcium imaging

HUVECs were plated on a thin-layered Matrigel-coated plate for 1 h to allow cells to attach. The cells were then loaded with Calcium 6-QF (Molecular Devices) in a Minimum Essential Medium–Hanks’ balanced salt solution (HBSS; 1:1) for 30 min at 37 °C in a CO_2_ incubator (32). The cells were washed gently with HBSS buffer with 2 mM Ca^2+^. The cells were imaged by using a Zeiss Axio observer Z1 with a 20× objective, and images were captured at 400-ms intervals. HBSS buffer with 2 mM Ca^2+^ was used during imaging. The intracellular calcium elevation of individual cells was analyzed with MetaMorph software (Molecular Devices). Cells overloaded with dye or only faintly fluorescent were excluded from analysis. The chemicals used in calcium imaging experiments included Yoda1 (Tocris; 5586), GsMTx4 (Abcam; ab141871), 5,6- eicosatrienoic acid (Santa Cruz Biotechnology; sc-221066), arachidonic acid (Sigma; A3611), HC067047 (Tocris; 4100), GSK1016790A (Sigma; G0798), AACOCF3 (Tocris; 1462), and YM26734 (Tocris; 2522).

### Monocyte-endothelial cell adhesion assay

To evaluate Yoda1-induced monocyte (THP-1 cells) adhesion to HUVECs, confluent HUVEC monolayers were pretreated with dimethyl sulfoxide (vehicle control), Yoda1 (1 μM), Yoda1 (1 μM) + HC067 (1 μM), or tumor necrosis factor-alpha (10 ng/ml), (Cell Biolabs, Inc; Catalog; CBA210) for up to 10 h in a humidified incubator at 37 °C and 5% CO_2_. After treatment, the media were removed and THP-1 cells were added to HUVEC monolayers for 1 h at 37 °C to allow attachment (70). Before THP-1 cells were added to HUVEC monolayers, the THP-1 cells were harvested and prepared in a cell suspension at 1.0 × 10^6^ cells/ml in serum-free media and loaded with LeukoTracker (a live cell imaging dye) from (Cell Biolabs, Inc; CBA210) as per the manufacturer’s instructions. The nonattached THP-1 cells were cleared by gently washing three times with serum-free media. The remaining adherent THP-1 cells loaded with LeukoTracker dye were imaged using a Zeiss Axio observer Z1 with a 4× objective. Adherent cells were counted using FIJI software. The average number of THP-1 cells was calculated from five image frames. The data presented are representative of three independent experiments.

### Immunofluorescent staining

HUVEC cells were washed with PBS (pH 7.4) and then fixed with 4% paraformaldehyde for 10 min at room temperature. The fixed cells were treated with 0.1% Triton X-100 in PBS (pH 7.4) for 15 min at room temperature and then blocked with 1% bovine serum albumin for 1 h at room temperature. Cells were incubated with antibodies against VE-cadherin (Santa Cruz Biotechnology; sc9989) overnight at 2 to 8 °C or antibodies against VCAM1 (Abcam; ab134047) for 1 h at room temperature (71). Secondary goat anti-rabbit or secondary goat anti-mouse IgG Alexa Fluor 594 (Jackson ImmunoResearch) was used after each step for 1 h at room temperature. Immunostaining of F-actin was performed on fixed cells using Alexa Flour 488 Phalloidin (Thermo Fisher scientific; A12379) for 1 h at room temperature. Cells were then washed three times with PBS for 10 min and incubated with Nunc Blue (Thermo Fisher scientific; R37606) and then mounted onto microscope slides with ProLong Gold antifade reagent (Invitrogen). Images of immunostained cells were taken with a Zeiss Axio observer Z1 with a 40× objective or a Leica SP5 inverted confocal microscope with a 40× oil objective. The images were processed initially with MetaMorph software (Molecular Devices) or Leica LAS AF lite software, and then the intensity of F-actin and width of VE-cadherin were measured with ImageJ software (11). To measure the F-actin fiber distribution, we used the plot profile from the analysis section of the ImageJ software (25). A line was drawn at the widest part of the cell perpendicular to the F-actin orientation across which the intensity of phalloidin 488 was measured. The length of the line drawn perpendicular to F-actin orientation was calculated by converting the length from pixel to μm (1 pixel = 0.1570 μm) according the Cool SNAP ES2 camera (Photometrics) specification with a 40× objective.

### Shear stress experiments

Laminar flow shear stress was generated using parallel-plate fluid flow chambers from Ibidi, GmbH. Two types of flow chambers were used in the experiments. A μ-Slide I 0.4 Luer flow chamber was used to achieve 12 dyne/cm^2^ or lower shear stress. A μ-Slide I 0.2 Luer flow chamber was used for forces of 25 dyne/cm^2^ (17, 33). The constant fluid flow rate with shear stress (τ) was determined as follows: τ = η χ 104.7 φ for μ-Slide I 0.4 Luer and τ = η χ 330.4 φ for μ-Slide I 0.2 Luer, where η = viscosity of the medium and φ = flow rate (according to the manufacturer’s instructions, Ibidi).

### Statistical analysis

Results are expressed as mean ± SD. Mean differences between 2 groups were analyzed by 2-tailed Student’s *t* test, and mean differences between multiple groups were analyzed by 1-way ANOVA with Tukey’s multiple comparison post test (GraphPad Prism 8). *p* values of less than 0.05 were considered significant, and ‘ns’ represents nonsignificant.

## Data availability

All the data described are contained within the manuscript and associated supporting information.

## Conflict of interest

The authors declare that they have no conflicts of interest with the contents of this article.
