# Supplementary material for: Piezo1 acts upstream of TRPV4 to induce pathological changes in endothelial cells due to shear stress
Source: J Biol Chem. 2020 Dec 14;296:100171. doi: 10.1074/jbc.RA120.015059 (PMC7948745; doi:10.1074/jbc.RA120.015059)
Supplement: Figures S1 and S2 [file mmc2.docx]

**Supporting Information**

**Piezo1 acts upstream of TRPV4 to induce pathological changes in endothelial cells due to shear stress**

Sandip M. Swain^1^ and Rodger A. Liddle^1,2^

Department of Medicine, Duke University^1^ and Department of Veterans Affairs Health Care System^2^, Durham, North Carolina 27710

Corresponding author:

Rodger A. Liddle, M.D.

Box 103859

1033A Genome Science Research Building 1

905 LaSalle Street

Duke University Medical Center

Durham, NC 27710

Telephone: (919) 681-6380

Email: [rodger.liddle@duke.edu](mailto:rodger.liddle@duke.edu)

**Running title**: Piezo1 and TRPV4 in endothelial cells

**Keywords:** Piezo1, TRPV4, endothelial cell, shear stress, calcium elevation, adherens junctions, phospholipase A2, actin remodeling

**Supporting figures and legends:**

**
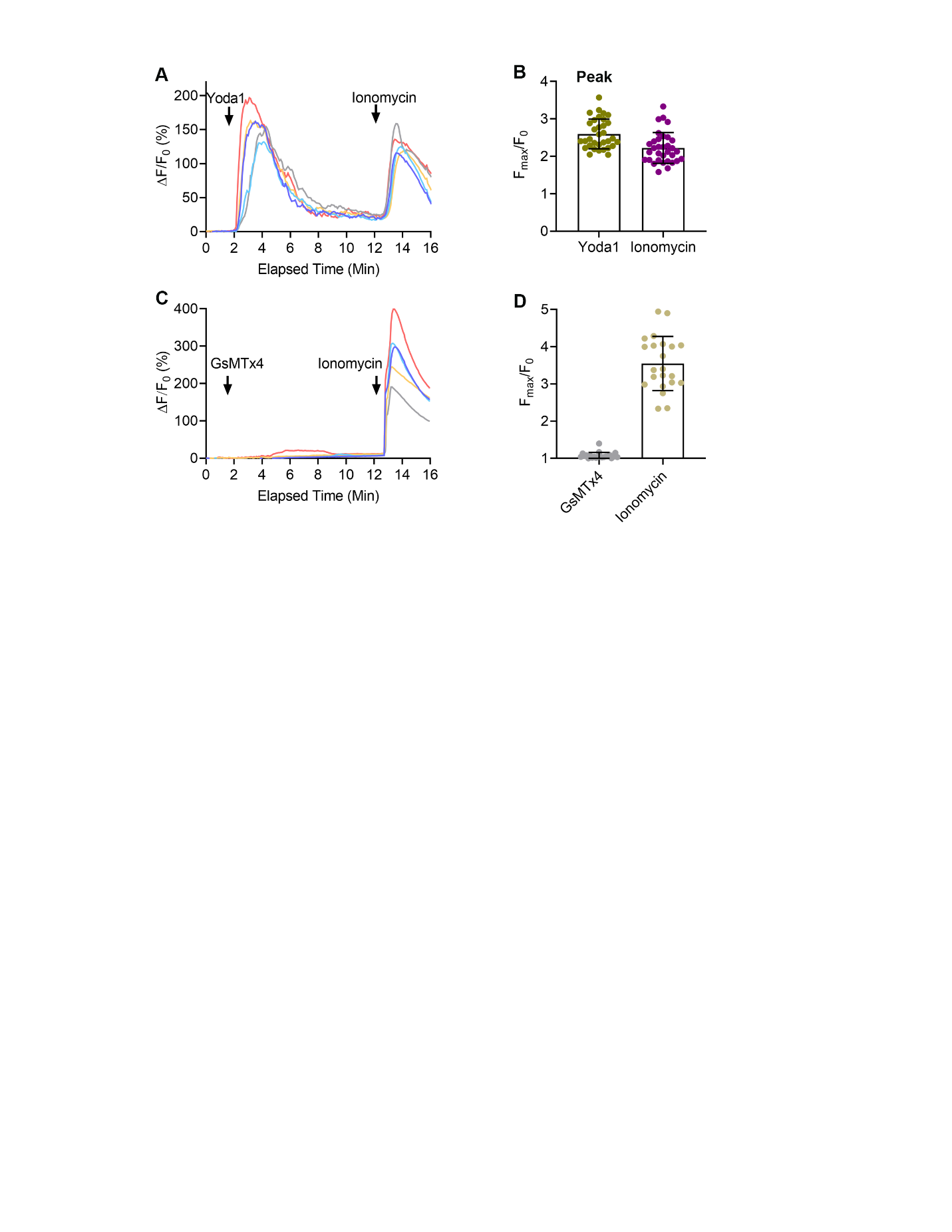
**

**Sup-Figure 1.** **Response of HUVECs to ionomycin following Yoda1 and GsMTx4**. (A) The relative fluorescence intensity (ΔF/F_0_) of calcium dye overtime with Yoda1 (10 µM) and ionomycin (1 µM) applied at different times. Black arrows show the time stimuli were applied. (B) The average maximum peak [Ca^2+^]_i_ intensity caused by Yoda1 and ionomycin from 33 cells. (C and D) Calcium influx with ionomycin (1 µM) after GsMTx4 (10 µM), (C) the relative fluorescence intensity of calcium dye overtime with GsMTx4 (10 µM) and ionomycin (1 µM) applied at different times marked with balck arrows, and (D) the average maximum peak [Ca^2+^]_i_ intensity caused by GsMTx4 and ionomycin from 22 cells.

**
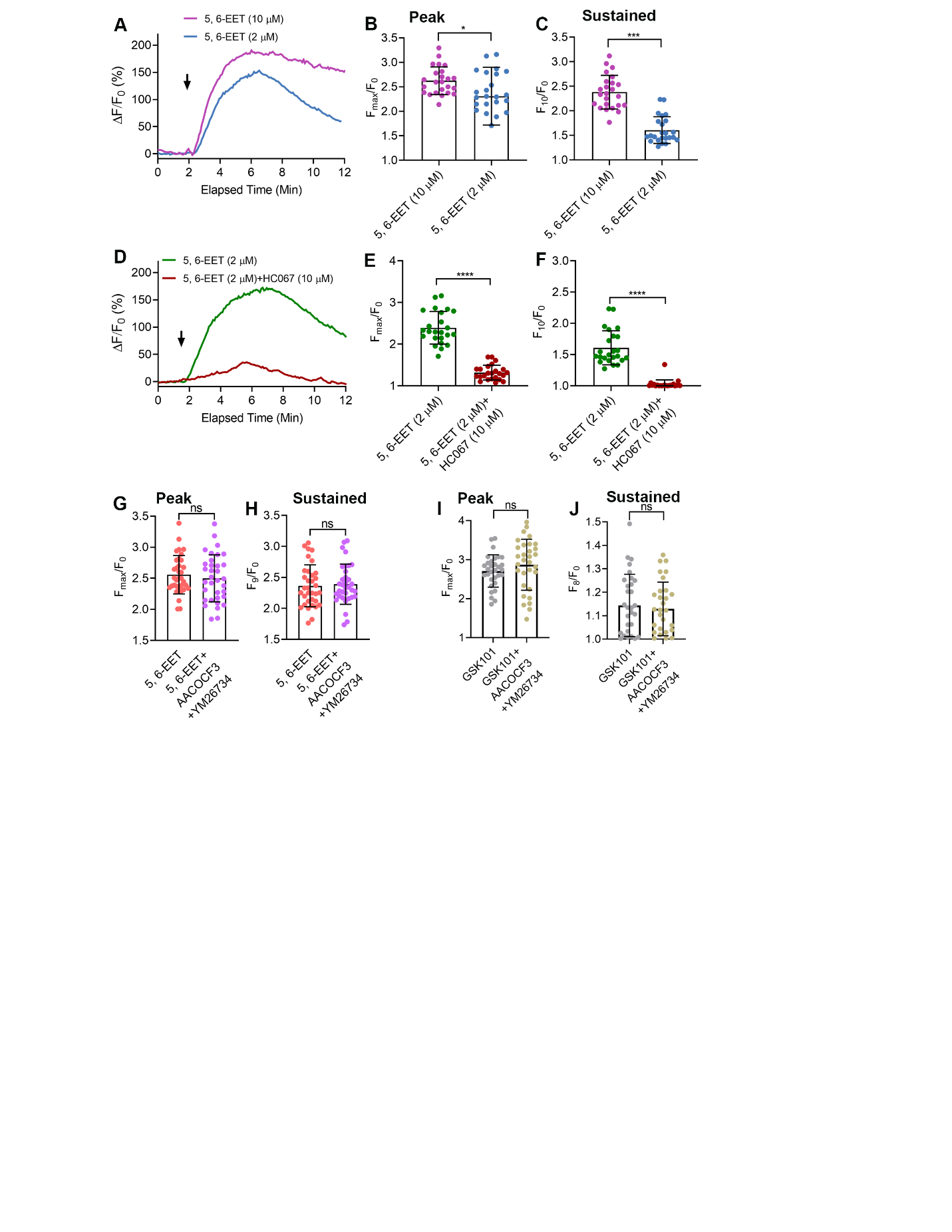
**

**Sup-Figure 2.** **TRPV4 antagonist, HC067, inhibits 5, 6-EET mediated-sustained [Ca^2+^]_i_  elevation in HUVECs.** (A) The relative fluorescence intensity (ΔF/F_0_) of calcium dye overtime with 2 µM and 5 µM 5, 6-EET. (B and C) The average maximum peak [Ca^2+^]_i_ intensity and the average sustained [Ca^2+^]_i_ intensity calculated at 10 min from 24 cells. (D) 5, 6-EET (2 µM)-mediated relative fluorescence intensity (ΔF/F_0_) of calcium dye overtime with and without HC067 (10 µM). (E and F) The average maximum peak [Ca^2+^]_i_ intensity and the average sustained [Ca^2+^]_i_ intensity calculated at 10 min from 24 cells. Black arrows show the time stimuli were applied. (G and H) The effects of PLA2 blockers, AACOCF3 (30 μM) and YM26764 (10 μM) on 5, 6-EET (5 µM)-induced peak [Ca^2+^]_i_ rise and sustained [Ca^2+^]_i_ rise measured at 9 min from 34 cells. (I and J) The effects of PLA2 blockers, AACOCF3 (30 μM) and YM26764 (10 μM) on GSK101 (100 nM)-induced maximum peak [Ca^2+^]_i_ rise and sustained [Ca^2+^]_i_ rise measured at 8 min from 31 cells. Statistical analyses were performed using two-tailed Student’s t test. Statistical analyses were performed using 1-way ANOVA with Tukey’s multiple comparisons. *P ≤ 0.05; *** P ≤ 0.001; ****P ≤0.0001. Data are shown as mean ± SD.

**Sup-Figure 1.** **Response of HUVECs to ionomycin following Yoda1**. (A) The relative fluorescence intensity (ΔF/F_0_) of calcium dye overtime with Yoda1 (10 µM) and ionomycin (1 µM) applied at different times. Black arrows show the time stimuli were applied. (B) The average maximum peak [Ca^2+^]_i_ intensity caused by Yoda1 and ionomycin from 33 cells.
